# Integrated analysis of rectal mucosal microbiome and transcriptome reveals a distinct microenvironment among young MSM

**DOI:** 10.1172/jci.insight.181720

**Published:** 2024-11-08

**Authors:** Cassie G. Ackerley, S. Abigail Smith, Phillip M. Murray, Praveen K. Amancha, Vanessa E. Van Doren, Gregory K. Tharp, Robert A. Arthur, Rama R. Amara, Yi-Juan Hu, Colleen F. Kelley

**Affiliations:** 1 The Hope Clinic of the Emory Vaccine Research Center, Division of Infectious Disease, Department of Medicine, and; 2 Department of Pediatrics, Emory University School of Medicine, Atlanta, Georgia, USA.; 3 Alexion Pharmaceuticals, Seaport, Massachusetts, USA.; 4 Non Human Primate Genomics Core, Emory National Primate Research Center,; 5 Emory Integrated Computational Core,; 6 Emory National Primate Research Center, and; 7 Department of Microbiology and Immunology, Emory University, Atlanta, Georgia, USA.; 8 Department of Biostatistics and Bioinformatics, Rollins School of Public Health, Emory University, Atlanta, Georgia, USA.

**Keywords:** Immunology, Microbiology, Adaptive immunity, Innate immunity, Transcription

## Abstract

Crosstalk between the microbiome and gut mucosa–resident immune cells plays a pivotal role in modulating immune responses to pathogens, including responses to HIV infection. However, how these interactions may differ between young men who have sex with men (YMSM) disproportionately impacted by HIV, as compared with older adult MSM (AMSM), is not well understood. A broad analysis of associations between the microbiome and rectal transcriptome revealed 10 microbial families/genera correlated with immunologic gene pathways. Specifically, the rectal transcriptome of YMSM was characterized by upregulation of T cell activation/differentiation pathways and signaling from multiple cytokine families compared with AMSM. The microbiome of YMSM was enriched with pathogenic genera, including *Peptostreptococcus*, shown to be positively correlated with type I IFN pathways important for antiviral immunity. These findings demonstrate that YMSM have a unique immune phenotype and rectal microenvironment and support further evaluation of biological factors that influence rectal HIV transmission.

## Abstract 2

The rectal mucosal transcriptomic profile and microbiome composition of young men who have sex with men suggests a unique immune phenotype that could impact rectal HIV transmission.

## Introduction

Men who have sex with men (MSM) are disproportionately at risk for HIV infection in the United States, with an estimated lifetime risk of 1 in 6 compared with 1 in 524 among men who have sex with women (1). Young MSM (YMSM) are a highly impacted group in the United States, with 24% of new HIV diagnoses among all MSM in 2020 occurring in males between the ages of 13 and 24 years (2). In an observational cohort study from 2015 in Bangkok, Thailand, MSM younger than 21 years were found to be at substantially higher risk of HIV infection compared with the older MSM cohorts. The YMSM were more likely to report drug use and engaging in receptive anal intercourse (RAI), yet all of the participating MSM reported similar frequency of condom use (3). In an analysis of per-contact risk estimation of men engaging in unprotected receptive anal sex with partners living with HIV, the per-contact risk for YMSM (<25 years) was higher compared with the older MSM (>30 years) despite a lower mean number of reported sexual contacts (7.1 vs. 10.3) (4). Thus, while individual factors, including number of sexual partners and types of sexual contact, certainly contribute to the likelihood of acquiring HIV, these findings suggest that there may also be biological factors that influence mucosal HIV transmission among YMSM.

Many studies to date that evaluate gut mucosal immunity and microbiome characteristics within the context of HIV acquisition risk combine all MSM into a single group for analyses, perhaps with the unintended result of overlooking distinct immune phenotypes influenced by age, sexual behaviors, or other potentially important demographic and lifestyle factors. In a comparison of the rectal mucosal environment of HIV-negative YMSM (18–21 years) and older adult men who have sex with men (AMSM; ≥35 years), YMSM had higher frequencies of activated Ki67^+^CD4^+^ memory T cells, a distinct microbiome composition, and higher HIV viral replication ex vivo in rectal tissues compared with AMSM (5). These findings indicate that the immune phenotype of YMSM may portend a greater susceptibility to HIV transmission within the rectal mucosa (RM) compared with older MSM.

Crosstalk between the gut microbiota and host mucosal immune system helps to maintain gut barrier integrity, intestinal motility, immune regulation, and inflammation homeostasis (6). Numerous factors have been shown to influence changes in the microbiome, including age and sexual practices. While gut microbiome shifts in the early and late stages of life have received considerable attention (7, 8), there is evidence that adolescents and adults have distinct microbiome compositions, with *Clostridium* and *Bifidobacterium* spp. being enriched among adolescents compared with adults (9). Multiple studies from our group and others have demonstrated that MSM have an altered microbiome composition typified by an increased *Prevotella*-to-*Bacteroides* ratio that may be associated with a proinflammatory rectal mucosal immune environment (5, 10, 11). Yet, it remains challenging to determine how the predominance of certain bacteria within the gut may be directly or indirectly influencing mucosal immune responses. One approach to exploring associations between the mucosal immune environment and gut microbiome composition is to evaluate correlations between the rectal tissue transcriptome and microbiome datasets. The field of oncology has been at the forefront of utilizing this methodology to identify associations between the microbiome and transcriptomic profiles from cancerous tumors to better predict clinical outcomes (12, 13). Likewise, these methods also have the potential to advance HIV prevention research by elucidating the interactions between the gut microbiome and host transcriptome and its subsequent impact on HIV transmission.

In the current study, we endeavored to further expand our investigation to evaluate differences in the rectal mucosal transcriptomic profiles between YMSM and AMSM and to explore correlations between bacterial genera of interest and immunologic gene pathways enriched within the rectal tissues of these MSM cohorts. Furthermore, we aimed to better characterize gut microbes that have a strong association with mucosal immunity by assessing the correlations between the rectal transcriptome and microbiome profiles of all MSM included in this study.

## Results

### YMSM are younger at time of sexual debut and report a reduced frequency of RAI compared with older adult MSM.

Twenty-five YMSM (18–21 years) and 31 AMSM (35–65 years) were enrolled and underwent mucosal secretion and rectal biopsy sampling. Demographic and clinical characteristics are presented in Table 1. The YMSM were overall younger at time of sexual debut compared with AMSM (median 18 vs. 23 years of age), and YMSM reported fewer lifetime partners and a lower frequency of RAI encounters over the prior 12 months compared with the older males.

### The rectal mucosal transcriptomic profile of YMSM demonstrates enrichment of immunologic pathways important for T and B cell responses and cytokine signaling in comparison with AMSM.

**Figure 1:**
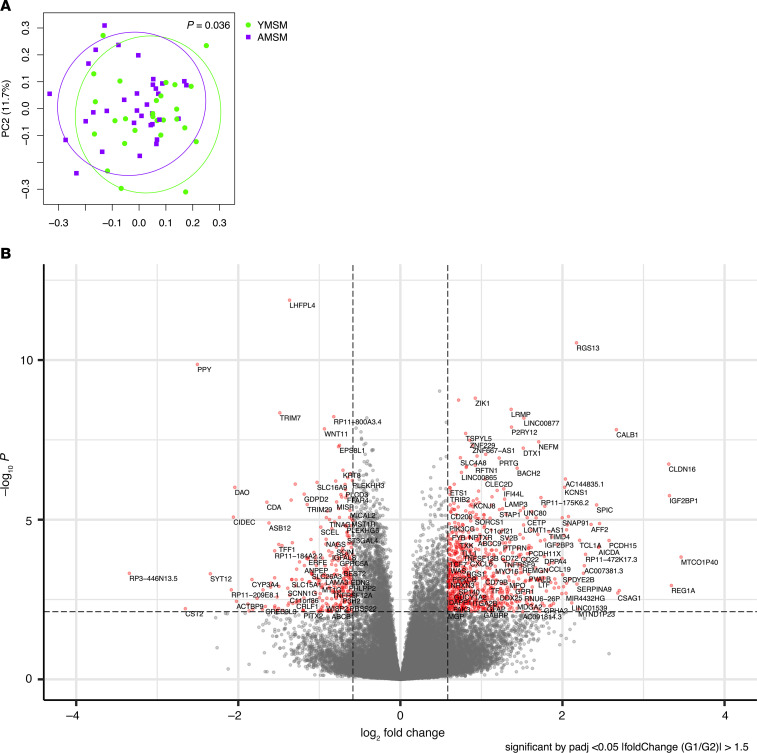
A comparison of rectal mucosal gene expression between YMSM and AMSM. (**A**) Principal component analysis illustrating distinct rectal mucosal RNA transcriptomic profiles for YMSM (green) and AMSM (purple) (*P* = 0.036, LDM). (**B**) Volcano plot demonstrating differential rectal mucosal gene expression between MSM cohorts. MSM, men who have sex with men; YMSM, young MSM; AMSM, adult MSM; LDM, linear decomposition modeling.

**Figure 2:**
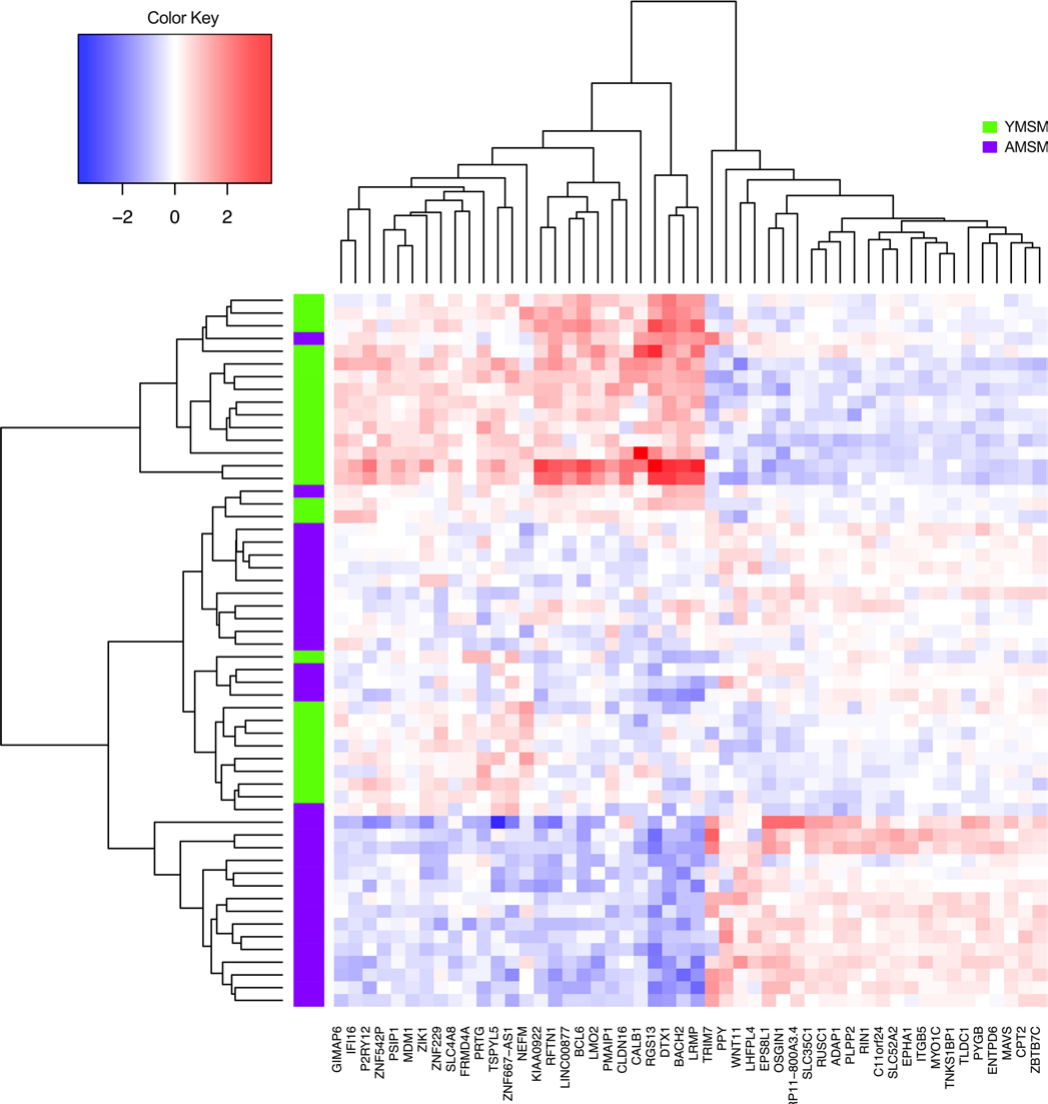
Heatmap of the top 50 genes differentially expressed between YMSM and AMSM based on *P* value significance. Rows labeled in green represent data from YMSM. Rows labeled in purple represent data from AMSM. YMSM, young men who have sex with men; AMSM, adult men who have sex with men.

To characterize differences in RM gene expression among YMSM compared to AMSM, transcriptional profiling was performed utilizing the RM tissue specimens from 25 YMSM and 31 AMSM. In a comparison of global differences in the RM transcriptome between study cohorts, distinct clustering of the transcriptome from YMSM was observed compared with AMSM in a 2-dimensional principal component analysis (PCA) plot (Figure 1A; linear decomposition modeling [LDM] *P* = 0.036). Differentially expressed genes (DEGs) were classified based on adjusted *P* values (≤0.05) and fold change in gene expression (log_2_FC ± 0.58). Of the 970 DEGs identified from the rectal transcriptome of YMSM compared with AMSM, 694 genes were upregulated and 276 genes were downregulated (Figure 1B). After filtering based on the adjusted *P* values, the top 50 DEGs among YMSM compared with AMSM demonstrated upregulation of genes associated with lymphocyte development (*LRMP*), T and B cell immune responses (*RFTN1*, *BACH2*), and downregulation of genes involved in phagocytosis of pathogens (*MYO1C*) and antigen processing/cross presentation (*ITGB5*) (Figure 2).

**Figure 3:**
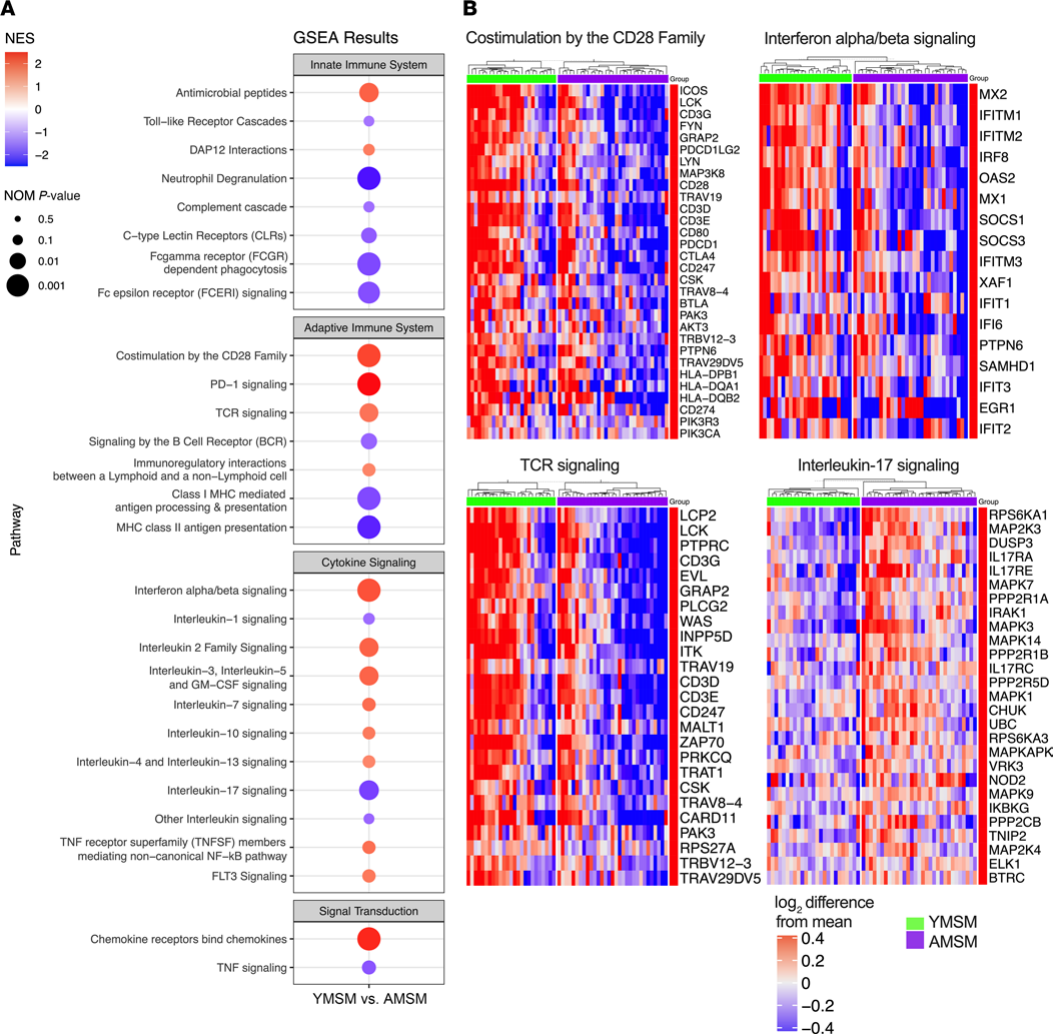
Immunologic pathway analysis based on differential gene expression between YMSM and AMSM. (**A**) Dot plot showing immunologic pathways enriched in YMSM compared with AMSM based on GSEA results using the Reactome gene set. (**B**) Heatmaps of the leading-edge genes from select pathways determined to be enriched by GSEA. Blue denotes downregulation, whereas red denotes upregulation. GSEA, gene set enrichment analysis; NES, normalized enrichment score; NOM, nominal.

Next, gene set enrichment analysis (GSEA) allowed for the identification of Reactome biological pathways (5% false discovery rate [FDR]) enriched among YMSM compared with AMSM (Figure 3A). Among all pathways associated with the innate immune system, the rectal transcriptomic profile of YMSM demonstrated enrichment of pathways associated with antimicrobial peptides and DNAX-activation protein 12 (DAP12) interactions (an adaptor protein that mediates intracellular signaling for monocytes and macrophages and is involved in promoting the expression of proinflammatory cytokines) (14). In contrast, there was downregulation of gene pathways associated with neutrophil degranulation, Fcγ receptor–dependent (FCGR-dependent) phagocytosis, and Fcε receptor (FCERI) signaling among YMSM. Pathways analysis also revealed enrichment of adaptive immune–related pathways, including costimulation by the CD28 family, programmed death-1 (PD-1) signaling, and T cell receptor (TCR) signaling among YMSM (Figure 3B). The pathways involving MHC class I and II antigen presentation were both downregulated in YMSM compared with AMSM. The tissues from YMSM showed enrichment in numerous cytokine family signaling pathways, including IFN-α/β signaling, IL-2, IL-3, IL-4, IL-5, IL-7, IL-10, IL-13, and granulocyte macrophage colony–stimulating factor (GM-CSF). IL-1 and IL-17 pathways were downregulated in YMSM compared with AMSM. Finally, the signal transduction pathway involving chemokine receptors binding to chemokines was also enriched among YMSM compared with AMSM.

### Enrichment of Peptostreptococcus and lower relative abundance of Eubacterium halli group among YMSM are correlated with cytokine signaling pathways involved in rectal mucosal immune responses.

For this study, we compared α and β diversity measures between YMSM and AMSM. Similar to our previous findings with slightly larger cohorts (5), we found no difference in α diversity measured by the Shannon Index between groups (*P* = 0.22; Supplemental Figure 1A; supplemental material available online with this article; https://doi.org/10.1172/jci.insight.181720DS1), yet we observed differences between groups in relative abundance of bacterial communities using the Bray-Curtis measurement (*P* = 0.015; Supplemental Figure 1B). In this analysis, there were no statistically significant differences in β diversity as measured by the Jaccard distance (*P* = 0.25; Supplemental Figure 1B). It is likely this result varies from our initial analysis with a larger cohort, as the Jaccard distance is useful in assessing differences in the presence or absence of less abundant taxa, and therefore, this measure is more likely to be sensitive to small changes in sample size. Notably, an assessment for homogeneity of dispersions showed no difference in variances between the 2 cohorts (*P* = 0.47).

In the primary microbiome analysis from our group, a comparison of the microbial composition between YMSM and AMSM demonstrated enrichment of *Prevotella*, *Peptostreptococcus*, *Peptoniphilus*, *Anaerococcus*, *Lawsonella*, and *Fusobacterium* among YMSM, and predominance of *Eubacterium hallii* group, *Olsenella*, *Ruminoclostridium.6*, *Negativibacillus*, and *Ruminococcaceae.UCG.009* among AMSM (5). To better understand the associations between these microbial amplicon sequence variants (ASVs) of interest and differentially expressed immunologic gene pathways in the rectal environment of MSM, we employed Pearson’s correlation method to test for associations between the microbiome and rectal transcriptome datasets from all 56 MSM, utilizing an FDR of 5%. For each of the microbial ASVs that were differentially enriched between YMSM and AMSM in our prior analysis, we grouped positively and negatively associated genes and performed downstream pathway analyses using the Reactome database (15) for gene sets with 5 or more genes (Table 2). Two ASVs, *Peptostreptococcus* and *Eubacterium hallii* group, were found to have significant correlations with genes involved in immunologic pathways. The relative abundance of *Peptostreptococcus* was positively correlated with IFN-α/β signaling and IFN-mediated antiviral immune pathways, while the relative abundance of *Eubacterium hallii* group was found to be negatively correlated with cytokine signaling pathways and with the noncanonical NF-κB pathway mediated by TNF receptor superfamily members. While the effects of microbiota on gut immune responses likely involve a complex array of both direct and indirect mediators, these findings suggest that certain microbial ASVs strongly impact immune cell signaling and antiviral immune responses in the rectum.

### Identification of microbiota-associated immunologic gene pathways from the rectal environment of MSM.

**Figure 4:**
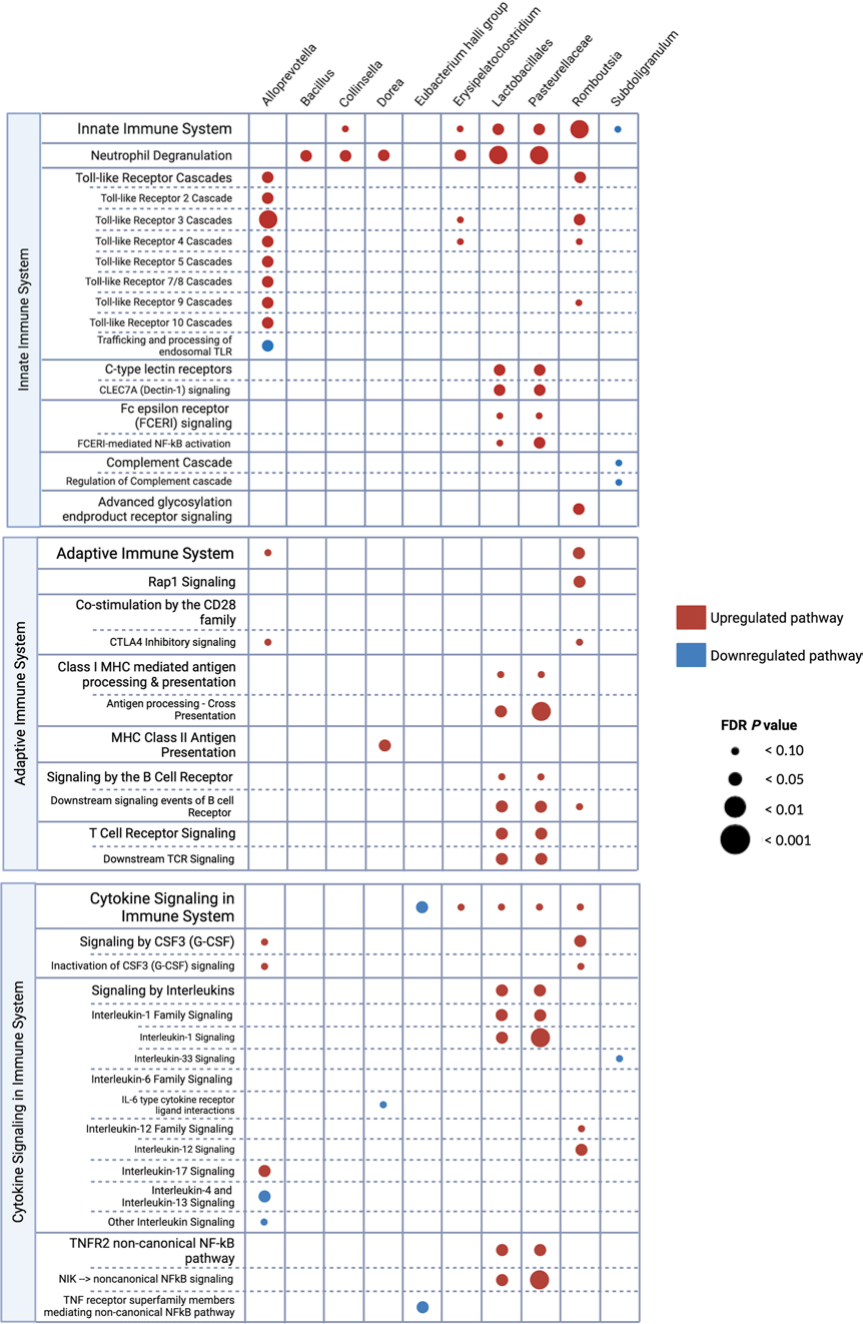
Immunologic pathway analysis of the microbe-associated genes. Dot plot of bacterial taxa identified as having associations with immunologic gene pathways based on Pearson’s correlation analyses. Figure was created with BioRender. FDR, false discovery rate; NF-κB, nuclear factor κB.

To explore the relationship between the microbiome and host rectal transcriptome of MSM, we examined associations between the relative abundance of individual microbial ASVs and transcriptomic gene expression (normalized enrichment score, NES) utilizing Pearson correlation coefficient testing. In an effort to identify highly relevant ASVs and to allow for robust downstream gene pathway analyses, we focused our attention on ASVs with 50 or more rectal transcriptome gene associations. A total of 12 ASVs were identified based on these criteria, and 10 microbial ASVs were found to have associations with genes implicated in immunologic gene pathways (Figure 4 and Supplemental Table 1). *Lactobacillales*, *Pasteurellaceae*, *Romboutsia*, and *Alloprevotella* were associated with upregulation of immunologic pathways involved in innate immunity, adaptive immunity, and cytokine signaling cascades. Higher relative abundance of *Alloprevotella* correlated with multiple Toll-like receptor (TLR) cascades, CTLA-4 inhibitory signaling, granulocyte colony–stimulating factor (G-CSF) signaling, and IL-17 signaling. Greater predominance of bacteria within the order of *Lactobacillales* and the family of *Pasteurellaceae* was associated with upregulation of pathways involving neutrophil degranulation, C-type lectin receptor activity, antigen processing and cross presentation, T and B cell receptor signaling, and IL-1 cytokine family signaling. Higher abundance of *Subdoligranulum* and *Eubacterium hallii* group correlated with downregulation of certain immunologic pathways, including complement cascade, IL-33 signaling, and TNF receptor signaling involved in the noncanonical NF-κB pathway. *Alloprevotella* abundance was found to be associated with downregulation of IL-4 and IL-13 signaling pathways.

## Discussion

YMSM are a subpopulation greatly impacted by HIV infection in the United States; thus, gaining a better understanding of their rectal immune phenotype and microbiome composition could allow for identification of biological factors that influence vulnerability to HIV acquisition for these young men. In this study, we identified a distinct rectal transcriptomic profile for YMSM characterized by enrichment of T cell–specific pathways and signaling from multiple cytokine families. We have previously shown that YMSM have a microbiome composition that differs from AMSM, and here, we find that 2 of these differentially abundant ASVs, *Peptostreptococcus* and *Eubacterium hallii* group, are associated with IFN-stimulated gene (ISG) and TNF receptor signaling pathways that are important for antiviral and antibacterial immunity. Finally, using a broader lens to explore correlations between the rectal transcriptome and gut microbiome from all MSM included in this study, we found 10 microbial ASVs that have strong associations with mucosal innate and adaptive immune responses.

In our comparative transcriptomic analysis between YMSM and AMSM, immunologic gene pathways involved in T cell activation, specifically CD28 costimulatory function, TCR signaling, and PD-1 signaling, were found to be upregulated in the rectal tissues from YMSM compared with AMSM. Additionally, there was corresponding upregulation of cytokine signaling pathways involved in T cell priming and cross presentation, including IFN-α/β and IL-2 signaling. These findings directly correspond to the cellular immune profile results previously published from this cohort of YMSM demonstrating greater frequencies of activated, proliferating Ki67^+^CD4^+^ memory T cells compared with AMSM (5). In another study from our group, we demonstrated that enrichment of immunologic pathways involved in T cell activation and differentiation from the rectal transcriptome of cisgender MSM prior to HIV exposure was associated with higher HIV viral replication following ex vivo rectal explant HIV challenge (16). As activated and proliferating CD4^+^ T cells are the prime target cells for HIV infection (17), this immune phenotype of YMSM suggests greater susceptibility to viral propagation following rectal HIV transmission.

The rectal transcriptomic profile of YMSM demonstrates an immune environment characterized by enrichment of type I IFNs, GM-CSF, and several interleukin signaling pathways. This cytokine milieu supports the development, proliferation, and survival of Th1 (IL-2, IL-7) and Th2 cells (IL-4, IL-5, IL-13) (18, 19). Increased GM-CSF within the tissues signals induction of granulocyte and macrophage populations (20), which are essential for the innate immune response. Prior studies have shown an increased frequency of CCR5-expressing macrophages in the distal rectum (21), and these cells are thought to serve as viral reservoirs that are capable of disseminating the virus to dendritic cells and T cells within the rectal mucosal tissues (22). Furthermore, we found enrichment of immunomodulatory mediators, specifically IL-10 and IL-3, within the rectum of these young men. IL-10 serves as an immunoinhibitory cytokine that helps to dampen the immune response (23), while IL-3 has been associated with sustaining T regulatory (Treg) cells within gut tissues to restore the balance of proinflammatory and antiinflammatory T cell responses (24). Secretion of these immunomodulatory cytokines, particularly IL-10, occurs concurrently with TCR activation and plays a critical role in maintaining gut homeostasis (23). While we are unable to fully evaluate the balance of proinflammatory and antiinflammatory mediators and their local tissue effects based on transcriptomic profiles alone, the overall rectal mucosal immune environment of these YMSM appears to be uniquely primed to respond to pathogens, particularly through cell-mediated immune responses. It is unclear whether this immune phenotype of YMSM, as compared with AMSM, is due to younger age, less frequent RAI, or possibly other unidentified factors. Based on our prior data showing higher frequencies of activated, proliferating CD4^+^ T cells within the rectal tissues of healthy YMSM, we hypothesized that more frequent RAI, as reported by the older MSM cohort in this study, may result in a higher degree of immune tolerance due to recurrent microabrasions and mucosal repair cycles (5). At present, the effects of RAI on mucosal immunity are not fully understood, and future studies are needed to differentiate the influence of sexual practices from other demographic, dietary, and behavioral factors when considering risk for HIV acquisition.

In addition to characterizing this distinct rectal transcriptomic profile in YMSM, we also sought to evaluate associations between immunologic gene pathways and the microbiome composition enriched in YMSM. The relative abundance of *Eubacterium hallii* group, an anaerobic gut commensal found to be less abundant among YMSM compared with AMSM, was negatively correlated with cytokine signaling and proinflammatory pathways involving the TNF receptor. Microbe-derived butyrate, a by-product of butyrate-producing bacteria, including those belonging to the *Eubacterium hallii* group, has been shown to induce Treg cell differentiation and proliferation of IL-10–producing T cells, thus suppressing colonic inflammation and maintaining immune homeostasis (25, 26). We also found increased relative abundance of *Peptostreptococcus* in this cohort of YMSM, which was positively correlated with type I IFN signaling pathways and the induction of ISGs. These immune mediators are critical for antiviral immunity and have been shown to modulate mucosal immune responses to HIV infection (27, 28). Notably, in prior studies evaluating the association between the penile foreskin microbiome and risk of HIV acquisition, *Peptostreptococcus* was identified as one of the pathogenic bacterial genera associated with higher cytokine production, local recruitment of susceptible CD4^+^ T cells, and an increased odds of HIV seroconversion (29, 30). An abundance of *Prevotella* and *Peptoniphilus*, two additional bacterial genera associated with increased odds of HIV acquisition at the penile mucosal site, also were found to be enriched in the gut microbiome composition of our YMSM cohort. As the immunologic effects of having a gut microbiome composition enriched with these anaerobic bacterial genera remains unclear, further investigation is warranted to better understand the relationship between microbiome composition, in particular the predominance of *Peptostreptococcus* and other pathogenic bacteria, and antiviral immune responses at mucosal sites following HIV exposure given the potential impact on HIV acquisition risk.

In this study, we used correlation analyses to evaluate for associations between immune signaling pathways and predominant microbial taxa in the rectal tissues of MSM. Butyrate-producing gut commensals, including *Subdoligranulum* and species within the *Eubacterium hallii* group, were found to be negatively correlated with certain innate immune pathways and IL-1 family signaling. These findings suggest that these gut commensals play an immunomodulatory role within the gut, which corresponds with existing literature describing butyrate as a microbial by-product that helps to maintain gut barrier integrity and limits proinflammatory cytokine production (31). Genera belonging to the order *Lactobacillales*, gut commensals commonly used in probiotic formulations, were associated with numerous immune processes involving innate and adaptive immune responses, including neutrophil activity, C-type lectin receptor signaling, antigen processing and cross presentation, and TCR and B cell receptor activity. Lactic acid producers, like *Lactobacillus* spp., have been shown to play an important role in maintaining gut homeostasis through strengthening intestinal barrier function, increasing production of antimicrobial peptides, and inducing secretory immunoglobulin A production (32). *Lactobacillus* also impacts gut immunity and inflammation by suppressing acute phase reactants (i.e., IL-6 and IL-8) that recruit immune cells into gut tissues, promoting T cell subset differentiation, and facilitating antiinflammatory cytokine release (33). The development of therapeutics that promote enrichment of gut commensals could positively impact the gut microbiota/immune axis and have the potential to modulate immune responses to various pathogens, including HIV.

*Alloprevotella*, a genus belonging to the family *Prevotellaceae*, was positively correlated with CTLA-4 inhibitory signaling, which regulates T cell proliferation, and IL-17 signaling. IL-17 is a critical cytokine mediator that modulates the interplay between gut commensals and epithelial cells and promotes proinflammatory immune responses and antibacterial function in the gut (34). Th17 effector cells are abundant in the mucosal-associated lymphoid tissues and serve as key producers of IL-17 cytokines. Importantly, these Th17 cells are primary target cells for HIV, expressing HIV coreceptors (CD4, α4β7, CCR5, and CXCR4), and thus play an important role in disseminating HIV during the acute phase of infection (35). In the literature, *Alloprevotella* has been shown to be enriched among people living with HIV (36) and has been identified as a potential predictor of high-grade intraepithelial squamous neoplasia when identified as a predominant anus-associated bacteria (37). While genera from the family *Prevotellaceae*, specifically *Prevotella*, *Paraprevotella*, and *Alloprevotella*, have previously been shown to be associated with RAI and HIV status, the findings from this study are among the first to our knowledge to describe specific mucosal immune pathways affected by their predominance in the microbiome composition.

One important limitation of this study is that we are examining the rectal immune environment and microbiome composition of healthy MSM at 1 cross-sectional point in time and in a presumed state of homeostasis. Thus, it is difficult to predict how these observed differences present in overall healthy individuals could impact immune responses following HIV exposure in vivo. Furthermore, we have included only MSM without concurrent sexually transmitted infections (STIs) in this study, which could impact the generalizability of these findings if these individuals have protective host factors, inherent or related to more frequent condom use, that may reduce their vulnerability to HIV infection. As described previously, many of the MSM in this study were found to have seropositivity to one or more herpes viruses (i.e., CMV, HSV-1, and HSV-2) (5). We are unable to exclude the possibility of viral shedding at time of rectal specimen collection, which would have the potential to influence the rectal transcriptomic immune profiles of the MSM cohorts in this study. Yet, this is of lower concern given participants were asymptomatic of gastrointestinal and genitourinary complaints at the time of rectal tissue biopsy sampling and based on prior data from our group demonstrating a very low incidence of HSV-2 viral shedding (2.4%) with anal PCR testing from healthy MSM participants at the time of the rectal biopsy procedure (10). Finally, these participants did not undergo anal cytology in this study. We have identified *Alloprevotella* in the microbiome composition among many of these MSM, and we do not know if this could be indicative of underlying atypical anal cytology, such as low- or high-grade intraepithelial squamous lesions. We anticipate that abnormal anal cytology would affect only a small minority of these participants, as many of them were quite young and all were asymptomatic without perianal genital lesions at the time of rectal tissue collection.

In summary, YMSM have a unique rectal mucosal immune environment with a transcriptomic profile characterized by CD4^+^ T cell activation and differentiation, the induction of macrophages, and enrichment of numerous cytokine signaling pathways. The microbiome composition of YMSM compared with AMSM is enriched with pathogenic genera, including *Prevotella*, *Fusobacterium*, and *Peptostreptococcus*, the latter of which is positively associated with type I IFN signaling pathways and has been identified as contributing to HIV acquisition risk at other mucosal sites (29, 30). Importantly, the results of this work oppose the assumption that MSM are a monolith in terms of the rectal mucosal microenvironment. It is more likely that age and sexual practices substantially influence rectal mucosal immunity and microbiome composition in ways that could make some individuals, particularly YMSM with earlier sexual debut and less frequent RAI, more susceptible to rectal HIV transmission. Furthermore, we have identified key bacterial families and genera that directly influence gut mucosal immune responses, which paves the way for identifying mechanistically how certain types of bacteria in the gut may facilitate or hinder mucosal antiviral immune responses during HIV transmission. Although our study has some limitations, it provides support for identifying demographic and lifestyle characteristics that may alter the gut microbiome and influence rectal mucosal immunity, as these factors likely have a direct impact on rectal HIV transmission risk and could influence the effectiveness of rectal microbicide products developed to prevent HIV infection.

## Methods

### Sex as a biological variable

Our study examined the rectal transcriptomic profile and microbiome composition from 2 cohorts of MSM. Participants assigned male sex at birth who engage in RAI were included in this study, as this population is disproportionately impacted by HIV infection in the United States. Samples were analyzed by cohort or pooled together for analysis, and sex was not considered as a biological variable.

### Clinical cohort

This study is a subanalysis of RNA transcriptomic and microbiome sequencing data from a larger cohort study designed to elucidate distinct features of the rectal mucosal environment of YMSM (18–21 years) following receptive anal sexual debut compared with AMSM (≥35 years) engaging in regular RAI, defined as at least 5 years with 12 or more episodes of RAI per year (5). All participants were healthy, HIV-negative, STI-negative cisgender men recruited from Atlanta, Georgia from August 2017 through January 2019. Eligibility criteria and clinical characteristics for each cohort have been previously described (5). Notably, none of the participants were using HIV preexposure prophylaxis. All participants presented for a screening visit that included informed consent, a medical history, physical exam, HIV and STI testing, and completion of a sexual health questionnaire. During a subsequent visit, microbiome swabs and rectal tissue biopsy specimens were collected via rigid sigmoidoscopy for subsequent 16S rRNA sequencing and tissue RNA sequencing, respectively. Following placement of the rigid sigmoidoscope into the rectum, mucosal secretions were collected using swabs and rectal tissue pinch biopsies were obtained using forceps from the same area within the rectum. All participants whose tissue and mucosal swab specimens yielded optimal transcriptomic and microbiome data were included in this substudy (*n* = 56).

### Tissue RNA sequencing

Two rectal tissue biopsies from each participant were stored in RNALater (Invitrogen, AM7021) at –80°C. The biopsies were homogenized in 350 μL Buffer RLT and RNA was extracted with on-column DNase digestion (Qiagen, RNeasy Micro kit). Following RNA quality assessment, 10 ng of total RNA was utilized for cDNA synthesis (Takara Bio, Clontech SMART-Seq v4 Ultra Low Input RNA kit). Amplified cDNA was then fragmented and appended with dual-indexed bar codes (Illumina, NexteraXT DNA Library Preparation kit), and libraries were validated with capillary electrophoresis (Agilent, TapeStation 4200), pooled at equimolar concentrations, and sequenced with yields of approximately 18 million reads per sample (Illumina HiSeq 3000, 100 single-read version). STAR version 2.5.2b (38) was used for alignment, transcripts were annotated using GRCh38 (https://www.ncbi.nlm.nih.gov/datasets/genome/GCF_000001405.26/), and abundance estimates were calculated using the htseq-count algorithm (38). Fifty-five out of 56 samples produced an RNA integrity number (RIN) of 8 or greater. The remaining sample had a moderate RIN score with a transcriptomic profile that was not deemed to be an outlier; therefore, this sample was included in downstream analyses.

### 16S rRNA sequencing and ASV clustering

16S rRNA sequencing from 56 mucosal secretion samples was performed on an Illumina MiSeq system using a MiSeq primer pair targeting the V3/V4 region (341 F [5′-CCTACGGGNGGCWGCAG-3′] and 805 R [5′-GACTACHVGGGTATCTAATCC-3′]). Mucosal secretion collection, DNA extraction, and 16S rRNA MiSeq sequencing were described previously (5). In brief, DNA was extracted using the Qiagen DNeasy Powersoil Kit (Qiagen, 12888) with 12.5 ng of DNA amplified using 16S Amplicon PCR Forward and Reverse Primers. Libraries were purified with Ampure XP beads (Beckman, A63880). Final 16S libraries were approximately 464 base pairs (bp) in length. For quality control, positive controls (*Escherichia coli* bacterial pellet) and negative controls (sterile water) were used to ensure appropriate extraction and to confirm the absence of contamination in extraction kit reagent. Additional controls (Zymo mock microbial community with known microbiome diversity; negative: sterile water) were used for the PCR amplification process. The quality of the libraries generated and included in this study were acceptable based on Illumina’s recommended guidelines (https://support.illumina.com/documents/documentation/chemistry_documentation/16s/16s-metagenomic-library-prep-guide-15044223-b.pdf). Raw sequences were demultiplexed using QIIME2 v2021.2 (39), and the Divisive Amplicon Denoising Algorithm 2 (DADA2) package (40) was used for error correcting and to create the feature table of ASVs within QIIME2. To ensure sequence uniformity, the first 30 bp were trimmed and reads were truncated at base 240. Final read counts for each sample are provided in Supplemental Table 2. Taxonomy was assigned using the Silva database (v132) (41) utilizing the qiime taxonomy modules with a nucleotide identity threshold of 97%. A threshold of 1000 reads was used for inclusion in downstream microbiome analyses.

### Statistics

Demographic and sexual behavior characteristics were compared between YMSM and AMSM cohorts using the Mann-Whitney *U* test for continuous variables and Fisher’s exact test for categorical variables.

#### Differential gene expression between cohorts.

RNA sequencing data were normalized with DESeq2 (42), and a batch variable was included as a covariate for batch correction. To compare global differences in the rectal mucosal transcriptome between YMSM and AMSM, PCA was used to visualize clustering of bulk transcriptomic data based on study cohort. LDM was then utilized to provide a *P* value for assessing the overall difference in gene expression between MSM cohorts and to identify the top 50 genes differentially expressed between these groups based on the *P* value significance. A more comprehensive evaluation of differential gene expression was performed between YMSM and AMSM using DESeq2 (42). Significant differential gene expression was determined based on a base mean expression of 20 or greater, log_2_FC ± 0.58, and adjusted *P* value of 0.05 or less. GSEA (43) was performed utilizing the Reactome geneset (44) in the MSigDB database (45).

#### Microbiome statistics.

Samples from all 56 MSM were included in the microbiome analyses. The α diversity, as measured by the Shannon Index, was compared between YMSM and AMSM cohorts using Wilcoxon’s rank-sum test. Microbiome composition dissimilarity was compared between cohorts using 2 β diversity metrics, Bray-Curtis and Jaccard distances. Global differences in β diversity measures between groups were calculated using the permutational multivariate analysis of variance (PERMANOVA) test, and a test for homogeneity of dispersions was also performed. A *P* value of less than 0.05 was used to indicate significance for comparisons in α and β diversity measures.

#### Microbiome and host gene expression associations.

For the rectal transcriptome data, the expression of each gene was first scaled to have zero mean and unit variance. Pearson’s correlation coefficient was used to test the association between the relative abundance of microbial ASVs and the NES of rectal transcriptome genes isolated from all 56 MSM participants. An FDR of 5% was used for significance. Based on our prior published findings, we first focused our attention on 11 out of 230 microbial ASVs identified as being differentially enriched (based on either relative abundance or presence/absence) among YMSM and AMSM. We evaluated the associations between these 11 ASVs and the rectal transcriptome dataset from all MSM. For each microbial ASV, positively and negatively correlated genes were grouped and gene sets with 5 or more genes were used for downstream Reactome pathway analyses (FDR *P* < 0.10). Next, we looked at associations between the rectal transcriptome and all remaining ASVs in our microbiome dataset. For this exploratory analysis, we set a more stringent parameter by evaluating microbial ASVs with 50 or more gene correlations identified. We concluded that these ASVs were most relevant given their abundance was highly associated with the rectal transcriptome and this cutoff allowed for robust downstream gene pathway analyses. Twelve microbial ASVs were identified based on these criteria. As above, positively and negatively correlated genes for each ASV were separately utilized as input for ReactomeFIViz pathway browser v3.7 (46). This allowed for the identification of significant (FDR *P* < 0.10) immunologic gene pathways associated with the relative abundance of microbial ASVs.

#### Statistical software.

All statistical analyses were performed in R package R4.3.2 (https://cran.r-project.org/bin/windows/base/old/4.3.2/). Graphs were prepared in R and Biorender (https://www.biorender.com/).

### Study approval

All participants provided written informed consent prior to participation in the study. The Emory University Institutional Review Board in Atlanta, Georgia approved the study procedures.

### Data availability

RNA-seq data were submitted to the NCBI GEO database (GSE270349: https://www.ncbi.nlm.nih.gov/geo/query/acc.cgi?acc=GSE270349). The 16S sequencing data included in this study are available in the NCBI Sequence Read Archive (SRA), accession number PRJNA881329.

## Author contributions

CFK is responsible for conception of the work, funding acquisition, oversight and conduct of the human participants’ protocol, oversight of the laboratory and data analysis and interpretation, and critical review of the manuscript. CGA performed data analyses and wrote the manuscript. PKA assisted with study design, performed laboratory assays, and provided critical review of the manuscript. SAS performed laboratory assays, contributed to data analyses, and provided critical review of the manuscript. PMM performed laboratory assays and provided critical review of the manuscript. YJH provided statistical expertise, contributed to the data analyses, and provided critical review of the manuscript. GKT, RAA, VEVD, and RRA contributed to the data analyses and provided critical review of the manuscript.

## Supplementary Material

Supplemental data

Supplemental table 1

Supporting data values

## Figures and Tables

**Table 1:**
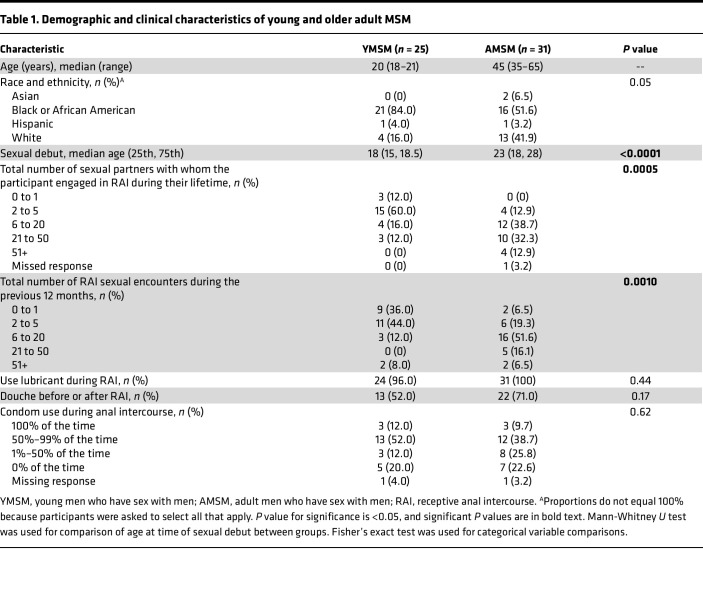
Demographic and clinical characteristics of young and older adult MSM

**Table 2:**
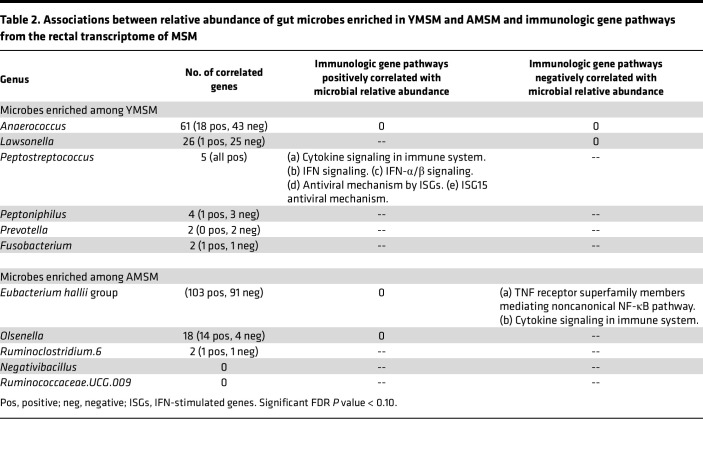
Associations between relative abundance of gut microbes enriched in YMSM and AMSM and immunologic gene pathways from the rectal transcriptome of MSM

## Version 1 10/01/2024

In-Press Preview

## Version 2 11/08/2024

Electronic publication

## References

[1] Hess KL (2017). Lifetime risk of a diagnosis of HIV infection in the United States. Ann Epidemiol.

[2] http://www.cdc.gov/hiv/library/reports/hiv-surveillance.html.

[3] Van Griensven F (2015). Temporal trends in HIV-1 incidence and risk behaviours in men who have sex with men in Bangkok, Thailand, 2006-13: an observational study. Lancet HIV.

[4] Scott HM (2014). Age, race/ethnicity, and behavioral risk factors associated with per contact risk of HIV infection among men who have sex with men in the United States. J Acquir Immune Defic Syndr.

[5] Ackerley CG (2022). The rectal mucosal immune environment and HIV susceptibility among young men who have sex with men. Front Immunol.

[6] Krishnamurthy HK (2023). Gut commensals and their metabolites in health and disease. Front Microbiol.

[7] Bosco N, Noti M (2021). The aging gut microbiome and its impact on host immunity. Genes Immun.

[8] Yao Y (2021). The role of microbiota in infant health: from early life to adulthood. Front Immunol.

[9] Agans R (2011). Distal gut microbiota of adolescent children is different from that of adults. FEMS Microbiol Ecol.

[10] Kelley CF (2017). The rectal mucosa and condomless receptive anal intercourse in HIV-negative MSM: implications for HIV transmission and prevention. Mucosal Immunol.

[11] Noguera-Julian M (2016). Gut microbiota linked to sexual preference and HIV infection. EBioMedicine.

[12] Li C (2021). Integrated analysis of microbiome and transcriptome data reveals the interplay between commensal bacteria and fibrin degradation in endometrial cancer. Front Cell Infect Microbiol.

[13] Huang H (2020). Integrated analysis of microbiome and host transcriptome reveals correlations between gut microbiota and clinical outcomes in HBV-related hepatocellular carcinoma. Genome Med.

[14] Li M (2023). Signaling pathways in macrophages: molecular mechanisms and therapeutic targets. MedComm (2020).

[15] Haw R (2020). Perform pathway enrichment analysis using ReactomeFIViz. Methods Mol Biol.

[16] Smith SA (2022). T-cell activation and B-cell interaction signatures in rectal tissues are associated with HIV replication in ex-vivo model of infection. AIDS.

[17] McKinnon LR, Kaul R (2012). Quality and quantity: mucosal CD4+ T cells and HIV susceptibility. Curr Opin HIV AIDS.

[18] Zhu J (2010). Differentiation of effector CD4 T cell populations (*). Annu Rev Immunol.

[19] Lee LF (2011). IL-7 promotes T(H)1 development and serum IL-7 predicts clinical response to interferon-β in multiple sclerosis. Sci Transl Med.

[20] Bhattacharya P (2015). GM-CSF: an immune modulatory cytokine that can suppress autoimmunity. Cytokine.

[21] McElrath MJ (2013). Comprehensive assessment of HIV target cells in the distal human gut suggests increasing HIV susceptibility toward the anus. J Acquir Immune Defic Syndr.

[22] Brown D, Mattapallil JJ (2014). Gastrointestinal tract and the mucosal macrophage reservoir in HIV infection. Clin Vaccine Immunol.

[23] Carlini V (2023). The multifaceted nature of IL-10: regulation, role in immunological homeostasis and its relevance to cancer, COVID-19 and post-COVID conditions. Front Immunol.

[24] Ullrich KA (2023). IL-3 receptor signalling suppresses chronic intestinal inflammation by controlling mechanobiology and tissue egress of regulatory T cells. Gut.

[25] Furusawa Y (2013). Commensal microbe-derived butyrate induces the differentiation of colonic regulatory T cells. Nature.

[26] Singh N (2014). Activation of Gpr109a, receptor for niacin and the commensal metabolite butyrate, suppresses colonic inflammation and carcinogenesis. Immunity.

[27] Utay NS, Douek DC (2016). Interferons and HIV infection: the good, the bad, and the ugly. Pathog Immun.

[28] Bosinger SE, Utay NS (2015). Type I interferon: understanding its role in HIV pathogenesis and therapy. Curr HIV/AIDS Rep.

[29] Prodger JL (2021). Penile bacteria associated with HIV seroconversion, inflammation, and immune cells. JCI Insight.

[30] Liu CM (2017). Penile anaerobic dysbiosis as a risk factor for HIV infection. mBio.

[31] Singh V (2022). Butyrate producers, “the sentinel of gut”: their intestinal significance with and beyond butyrate, and prospective use as microbial therapeutics. Front Microbiol.

[32] Dempsey E, Corr SC (2022). Lactobacillus spp. for gastrointestinal health: current and future perspectives. Front Immunol.

[33] Rastogi S, Singh A (2022). Gut microbiome and human health: exploring how the probiotic genus Lactobacillus modulate immune responses. Front Pharmacol.

[34] Brevi A (2020). Much more than IL-17A: cytokines of the IL-17 family between microbiota and cancer. Front Immunol.

[35] Renault C (2022). Th17 CD4+ T-cell as a preferential target for HIV reservoirs. Front Immunol.

[36] Lopera TJ (2021). A specific structure and high richness characterize intestinal microbiota of HIV-exposed seronegative individuals. PLoS One.

[37] Ron R (2021). Exploiting the microbiota for the diagnosis of anal precancerous lesions in men who have sex with men. J Infect Dis.

[38] Anders S (2015). HTSeq--a Python framework to work with high-throughput sequencing data. Bioinformatics.

[39] Bolyen E (2019). Reproducible, interactive, scalable and extensible microbiome data science using QIIME 2. Nat Biotechnol.

[40] Callahan BJ (2016). DADA2: high-resolution sample inference from Illumina amplicon data. Nat Methods.

[41] Quast C (2013). The SILVA ribosomal RNA gene database project: improved data processing and web-based tools. Nucleic Acids Res.

[42] Love MI (2014). Moderated estimation of fold change and dispersion for RNA-seq data with DESeq2. Genome Biol.

[43] Subramanian A (2005). Gene set enrichment analysis: a knowledge-based approach for interpreting genome-wide expression profiles. Proc Natl Acad Sci U S A.

[44] Fabregat A (2017). Reactome pathway analysis: a high-performance in-memory approach. BMC Bioinformatics.

[45] Liberzon A (2011). Molecular signatures database (MSigDB) 3.0. Bioinformatics.

[46] Wu G (2014). ReactomeFIViz: a Cytoscape app for pathway and network-based data analysis. F1000Res.

