# Supplementary material for: Integrated analysis of rectal mucosal microbiome and transcriptome reveals a distinct microenvironment among young MSM
Source: JCI Insight. 2024 Nov 8;9(21):e181720. doi: 10.1172/jci.insight.181720 (PMC11601568; doi:10.1172/jci.insight.181720)
Supplement: Supplemental data [file jciinsight-9-181720-s229.pdf]

Supplemental Material File

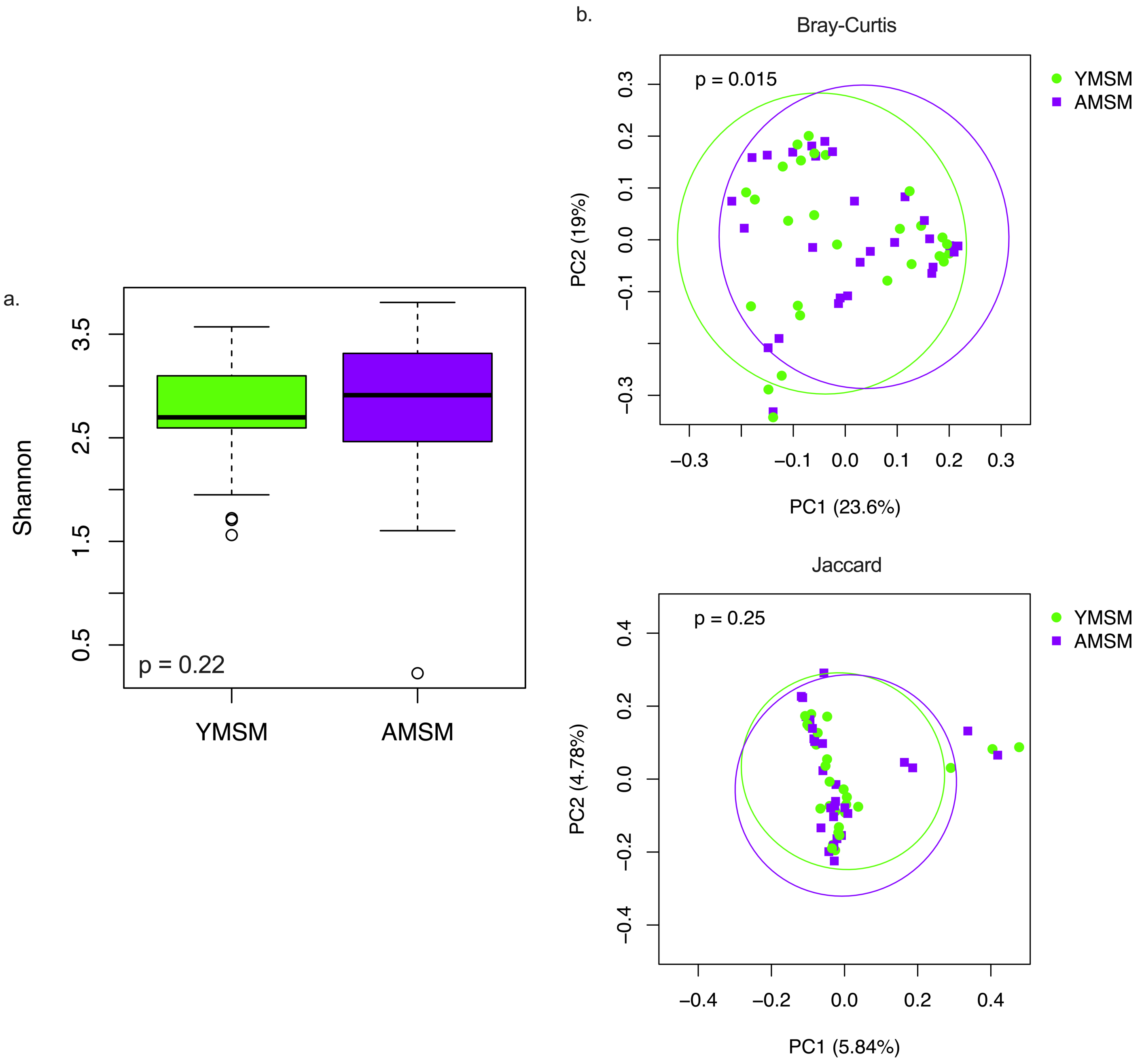

Figure S1. A comparison of alpha and beta diversity measurements between YMSM and AMSM cohorts. (a) Alpha diversity plot, estimated by the Shannon Index, shows no significant differences between groups. Boxes show first and third quartiles, whiskers illustrate values extending from the boxes up to ± 1.5x the interquartile range. **(b)** Principal coordinates analysis (PCoA) projection of Bray-Curtis and Jaccard distance between YMSM and AMSM.

Table S1: Correlations between Rectal Mucosal Microbiota and Transcriptome based on Immune Compartment

Pathway	ASV	# genes	FDR
**Innate Immune System**	Collinsella	40/1145	0.0873
**Innate Immune System**	Lactobacillales	11/1145	0.0166
**Innate Immune System**	Romboutsia	23/1145	0.0097036
**Innate Immune System**	Erysipelatoclostridium	103/1145	0.0672
**Innate Immune System**	Pasteurellaceae	11/1145	0.0116
**Innate Immune System**	Subdoligranulum	9/1145	0.0654
**Neutrophil Degranulation**	Collinsella	23/479	0.035
**Neutrophil Degranulation**	Bacillus	7/479	0.0366
**Neutrophil Degranulation**	Lactobacillales	9/479	0.0069079
**Neutrophil Degranulation**	Dorea	9/479	0.0387
**Neutrophil Degranulation**	Erysipelatoclostridium	52/479	0.0316
**Neutrophil Degranulation**	Pasteurellaceae	9/479	0.0053904
**Toll-like Receptor Cascades**	Romboutsia	6/155	0.0352
**Toll-like Receptor Cascades**	Alloprevatella	6/155	0.0196
**Trafficking and processing of endosomal TLR**	Alloprevatella	3/13	0.0266
**Toll Like Receptor 2 (TLR2) Cascade**	Alloprevatella	4/102	0.0497
Toll Like Receptor TLR1:TLR2 Cascade	Alloprevatella	4/102	0.0497
Toll Like Receptor TLR6:TLR2 Cascade	Alloprevatella	4/102	0.0497
**Toll Like Receptor 3 (TLR3) Cascade**	Erysipelatoclostridium	15/93	0.0511
**Toll Like Receptor 3 (TLR3) Cascade**	Romboutsia	5/93	0.0265
**Toll Like Receptor 3 (TLR3) Cascade**	Alloprevatella	6/93	0.0065373
TICAM1, RIP1-mediated IKK complex recruitment	Alloprevatella	2/19	0.0581
TICAM1, RIP1-mediated IKK complex recruitment	Romboutsia	2/19	0.0699
TICAM1, RIP1-mediated IKK complex recruitment	Erysipelatoclostridium	6/19	0.0453
**Toll Like Receptor 4 (TLR4) Cascade**	Alloprevatella	6/132	0.012
MyD88:MAL(TIRAP) cascade initiated on plasma membrane	Alloprevatella	4/102	0.0497
TRIF(TICAM1)-mediated TLR4 signaling	Alloprevatella	6/97	0.0065373
TRIF(TICAM1)-mediated TLR4 signaling	Romboutsia	5/97	0.0284
TRIF(TICAM1)-mediated TLR4 signaling	Erysipelatoclostridium	15/97	0.062
IKK complex recruitment mediated by RIP1	Alloprevatella	2/23	0.0714
IKK complex recruitment mediated by RIP1	Romboutsia	2/23	0.08
IKK complex recruitment mediated by RIP1	Erysipelatoclostridium	6/23	0.0712
My D88-independent TLR4 cascade	Erysipelatoclostridium	15/97	0.062
My D88-independent TLR4 cascade	Romboutsia	5/97	0.0284
My D88-independent TLR4 cascade	Alloprevatella	6/97	0.0065373
**Toll Like Receptor 5 (TLR5) Cascade**	Alloprevatella	4/85	0.0413
**Toll Like Receptor 7/8 (TLR7/8) Cascade**	Alloprevatella	4/91	0.0417
MyD88 dependent cascade initiated on plasma membrane	Alloprevatella	4/90	0.0414
TRAF6 mediated induction of NFkB and MAP kinases upon TLR7/8 or 9 activation	Alloprevatella	4/89	0.0414
IRAK1 recruits IKK complex upon TLR7/8 or 9 stimulation	Lactobacillales	1/10	0.0667
IRAK1 recruits IKK complex upon TLR7/8 or 9 stimulation	Pasteurellaceae	1/10	0.0552
TRAF6 mediated IRF7 activation in TLR7/8 or 9 signaling	Lactobacillales	1/9	0.0667
TRAF6 mediated IRF7 activation in TLR7/8 or 9 signaling	Pasteurellaceae	1/9	0.0552
TRAF6 mediated IRF7 activation in TLR7/8 or 9 signaling	Subdoligranulum	1/9	0.0958
**Toll Like Receptor 9 (TLR9) Cascade**	Romboutsia	4/94	0.0621
**Toll Like Receptor 9 (TLR9) Cascade**	Alloprevatella	4/94	0.042
**Toll Like Receptor 10 (TLR10) Cascade**	Alloprevatella	4/85	0.0413
MyD88 cascade initiated on plasma membrane	Alloprevatella	4/85	0.0413
MAP kinase activation	Romboutsia	3/63	0.0872
MAP kinase activation	Alloprevatella	4/63	0.0222
IRAK1 recruits IKK complex	Lactobacillales	1/10	0.0667
IRAK1 recruits IKK complex	Pasteurellaceae	1/10	0.0552
MAPK targets / Nuclear events mediated by MAP kinases	Erysipelatoclostridium	7/31	0.0751
MAPK targets / Nuclear events mediated by MAP kinases	Alloprevatella	3/31	0.0265
JNK (c-Jun kinases) phosphorylation and activation mediated by activated human TAK1	Lactobacillales	1/18	0.0704
JNK (c-Jun kinases) phosphorylation and activation mediated by activated human TAK1	Pasteurellaceae	1/18	0.0659

activated TAK1 mediates p38 MAPK activation	Lactobacillales	1/19	0.0742
activated TAK1 mediates p38 MAPK activation	Pasteurellaceae	1/19	0.0694
ERK/MAPK Targets	Erysipelatoclostridium	6/22	0.0663
ERK/MAPK Targets	Romboutsia	2/22	0.08
ERK/MAPK Targets	Alloprevatella	3/22	0.0154
ERKs are inactivated	Alloprevatella	3/13	0.0076056
ERKs are inactivated	Romboutsia	2/13	0.0476
**C-type lectin receptors (CLRs)**	Lactobacillales	3/144	0.0414
**C-type lectin receptors (CLRs)**	Pasteurellaceae	3/144	0.0344
CLEC7A (Dectin-1) signaling	Lactobacillales	3/100	0.0235
CLEC7A (Dectin-1) signaling	Pasteurellaceae	3/100	0.0194
Dectin-1 mediated noncanonical NF-kB signaling	Lactobacillales	3/62	0.0125
Dectin-1 mediated noncanonical NF-kB signaling	Pasteurellaceae	3/62	0.0102
**Fc epsilon receptor (FCERI) signaling**	Lactobacillales	3/210	0.0667
**Fc epsilon receptor (FCERI) signaling**	Pasteurellaceae	3/210	0.0552
FCERI mediated NF-kB activation	Lactobacillales	3/157	0.0517
FCERI mediated NF-kB activation	Pasteurellaceae	3/157	0.0431
**Advanced glycosylation endproduct receptor signaling**	Romboutsia	2/13	0.0476
**Complement Cascade**	Subdoligranulum	3/138	0.054
Regulation of Complement cascade	Subdoligranulum	3/127	0.054

Pathway	ASV	# genes	FDR
**Adaptive Immune System**	Alloprevatella	12/835	0.0805
**Adaptive Immune System**	Romboutsia	18/835	0.017
CTLA4 inhibitory signaling	Alloprevatella	2/21	0.0685
CTLA4 inhibitory signaling	Romboutsia	2/21	0.08
CD28 dependent PI3K/Akt signaling	Lactobacillales	1/22	0.0854
CD28 dependent PI3K/Akt signaling	Pasteurellaceae	1/22	0.08
**Class I MHC mediated antigen processing & presentation**	Lactobacillales	4/380	0.0675
**Class I MHC mediated antigen processing & presentation**	Pasteurellaceae	4/380	0.0552
**Antigen processing-Cross presentation**	Lactobacillales	4/109	0.0114
**Antigen processing-Cross presentation**	Pasteurellaceae	4/109	0.0092996
ER-Phagosome pathway	Lactobacillales	4/93	0.0114
ER-Phagosome pathway	Pasteurellaceae	4/93	0.0092996
Cross-presentation of soluble exogenous antigens (endosomes)	Lactobacillales	3/50	0.0114
Cross-presentation of soluble exogenous antigens (endosomes)	Pasteurellaceae	3/50	0.0092996
**MHC class II Antigen Presentation**	Dorea	5/108	0.0142
**Signaling by the B Cell Receptor (BCR)**	Lactobacillales	3/175	0.0667
**Signaling by the B Cell Receptor (BCR)**	Pasteurellaceae	3/175	0.0552
**Downstream signaling events of B Cell Receptor (BCR)**	Lactobacillales	3/81	0.0166
**Downstream signaling events of B Cell Receptor (BCR)**	Romboutsia	4/81	0.0512
**Downstream signaling events of B Cell Receptor (BCR)**	Pasteurellaceae	3/81	0.0128
Calcineurin activates NFAT	Romboutsia	2/9	0.0348
Activation of NF-kappa B in B cells	Lactobacillales	3/67	0.0129
Activation of NF-kappa B in B cells	Pasteurellaceae	3/67	0.0106
**TCR signaling**	Lactobacillales	3/126	0.0305
**TCR signaling**	Pasteurellaceae	3/126	0.0242
**Downstream TCR signaling**	Lactobacillales	3/104	0.0261
**Downstream TCR signaling**	Pasteurellaceae	3/104	0.0216
**Rap1 signalling**	Romboutsia	3/16	0.0122

Table S2: 16S Sequencing – Raw and Cleaned Read Counts

Sample Name	Forward Raw Read Count	Reverse Raw Read Count	Total Raw Read Count	Post-DADA2 Cleaning Total Read Count
CKE12524-70560-ARM-002	170744	170744	341488	130109
CKE12524-70560-ARM-003	126263	126263	252526	86840
CKE12524-70560-ARM-005	181941	181941	363882	131307
CKE12524-70560-ARM-006	166583	166583	333166	126513
CKE12524-70560-ARM-007	121177	121177	242354	62707
CKE12524-70560-ARM-012	135144	135144	270288	95014
CKE12524-70560-ARM-015	102262	102262	204524	77680
CKE12524-70560-ARM-017	15003	15003	30006	9356
CKE12524-70560-ARM-018	84161	84161	168322	54101
CKE12524-70560-ARM-019	111103	111103	222206	79978
CKE12524-70560-ARM-021	79879	79879	159758	46089
CKE12524-70560-ARM-022	97387	97387	194774	52882
CKE12524-70560-ARM-023	24153	24153	48306	16760
CKE12524-70560-ARM-024	90209	90209	180418	49173
CKE12524-70560-ARM-026	96203	96203	192406	49964
CKE12524-70560-ARM-028	9857	9857	19714	3410
CKE12524-70560-ARM-031	117491	117491	234982	88922
CKE12524-70560-ARM-032	112928	112928	225856	74528
CKE12524-70560-ARM-035	119225	119225	238450	93546
CKE12524-70560-ARM-038	91623	91623	183246	55727
CKE12524-70560-ARM-040	84789	84789	169578	57760
CKE12524-70560-ARM-042	97497	97497	194994	43954
CKE12524-70560-ARM-043	91381	91381	182762	53571
CKE12524-70560-ARM-045	113394	113394	226788	67638
CKE12524-70560-ARM-046	53004	53004	106008	15453
CKE12524-70560-ARM-047	102309	102309	204618	74165
CKE12524-70560-ARM-048	106802	106802	213604	58519
CKE12524-70560-ARM-051	59869	59869	119738	41803
CKE12524-70560-ARM-052	108951	108951	217902	78345
CKE12524-70560-ARM-053	37013	37013	74026	28062
CKE12524-70560-ARM-054	153771	153771	307542	114682
CKE12524-70560-ARM-056	178203	178203	356406	101641
CKE12524-70560-ARM-059	99617	99617	199234	59985
CKE12524-70560-ARM-065	106550	106550	213100	73814
CKE12524-70560-ARM-066	110535	110535	221070	74568
CKE12524-70560-ARM-068	113482	113482	226964	79590
CKE12524-70560-ARM-072	113139	113139	226278	76191
CKE12524-70560-ARM-073	98182	98182	196364	65731
CKE12524-70560-ARM-078	108564	108564	217128	64739
CKE12524-70560-ARM-081	127366	127366	254732	78633
CKE12524-70560-ARM-083	107561	107561	215122	64215
CKE12524-70560-ARM-088	84603	84603	169206	51065
CKE12524-70560-ARM-090	97256	97256	194512	53043
CKE12524-70560-ARM-093	101375	101375	202750	77216
CKE12524-70560-ARM-094	151247	151247	302494	107295
CKE12524-70560-ARM-095	98059	98059	196118	70650
CKE12524-70560-B1-Blank1	2487	2487	4974	1558
CKE12524-70560-B1-Blank2	1148	1148	2296	548
CKE12524-70560-B2-Blank1	1814	1814	3628	1020
CKE12524-70560-B2-Blank2	1812	1812	3624	1094
CKE12524-70560-B2-Blank3	2532	2532	5064	1707
CKE12524-70560-B2-Blank4	5912	5912	11824	3599
CKE12524-70560-B3-Blank1	822	822	1644	337
CKE12524-70560-B3-Blank2	1466	1466	2932	889
CKE12524-70560-B3-Blank3	2382	2382	4764	1430
CKE12524-70560-B3-Blank4	557	557	1114	240
CKE12524-70560-NTC-P3	1155	1155	2310	367
CKE12524-70560-NTC1-P1	4595	4595	9190	2565
CKE12524-70560-NTC2-P1	4540	4540	9080	2662
CKE12524-70560-STI-006	87213	87213	174426	54938
CKE12524-70560-STI-007	109710	109710	219420	63916
CKE12524-70560-STI-011	109509	109509	219018	65194
CKE12524-70560-STI-012	114639	114639	229278	64605
CKE12524-70560-STI-017	133090	133090	266180	104053
CKE12524-70560-STI-018	109826	109826	219652	66288

CKE12524-70560-STI-020	105127	105127	210254	65930
CKE12524-70560-STI-021	112438	112438	224876	67099
CKE12524-70560-STI-024	129766	129766	259532	64051
CKE12524-70560-STI-046	88654	88654	177308	65259
CKE12524-70560-ZYMO-P1	116032	116032	232064	77655
CKE12524-70560-Zymo1-P3	108829	108829	217658	72861
CKE12524-70560-Zymo2-P3	192822	192822	385644	126344
CKE12597-72615-B1Neg1	2514	2514	5028	1315
CKE12597-72615-B1Neg2	519	519	1038	108
CKE12597-72615-B1Pos1	240192	240192	480384	189014
CKE12597-72615-B2Neg1	4429	4429	8858	2152
CKE12597-72615-B2Neg2	3215	3215	6430	2166
CKE12597-72615-B2Pos1	232830	232830	465660	187320
CKE12597-72615-NTC-repeat	7836	7836	15672	5030
CKE12597-72615-NTC1	1781	1781	3562	882
CKE12597-72615-NTC2	1903	1903	3806	917
CKE12597-72615-Zymo-repeat	753945	753945	1507890	535016
CKE12597-72615-Zymo1	256334	256334	512668	182675
CKE12597-72615-Zymo2	258614	258614	517228	182797
